# The Patient Voice in Aesthetic Medicine: Findings From a Global Survey of Cosmetic Neurotoxin Patients

**DOI:** 10.1093/asjof/ojaf109

**Published:** 2025-10-13

**Authors:** Julia K Garcia, Mary Elizabeth Bennett, Sylwia Lipko-Godlewska, Terrence C Keaney, Susan L Hogue, Maria Musumeci

## Abstract

**Background:**

Patient satisfaction following cosmetic neurotoxin treatment has been widely studied, but little is known about how this affects downstream behavior.

**Objectives:**

To investigate the actions taken by patients following cosmetic neurotoxin injection, with regard to their most and least positive treatment experiences.

**Methods:**

This was an online, self-administered survey conducted among adults residing in Brazil, Canada, United Kingdom, and United States. Eligible participants had received 4 or more previous neurotoxin treatments to temporarily improve the appearance of upper facial lines, at least one of which was in the past 12 months.

**Results:**

A total of 1612 respondents completed the questionnaire (61% female; mean age: 38.1 ± 9.6 years). After their most positive experience, 81% said they engaged in actions directed toward their healthcare professional (HCP) (eg, scheduled another treatment or posted a review on the HCP's website), and 81% took actions directed at others (eg, talked to friends and family or posted a review online). After their least positive experience, 58% engaged in actions directed toward their HCP (with 22% expressing dissatisfaction directly), and 73% took actions to inform other people; many said they discouraged others from using their HCP (52%) or from seeking cosmetic neurotoxin injections altogether (27%).

**Conclusions:**

Respondents were less likely to inform their HCP of their satisfaction level after their least positive experience of cosmetic neurotoxin treatment compared with the most positive. Thus, practitioners may often be unaware of dissatisfied individuals. Patient-centered care and consistent proactive follow-up are essential to understanding patient perspectives on outcomes.

**Level of Evidence:**

5 (Therapeutic) 
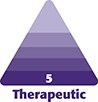

A patient-centered approach is essential to good practice with cosmetic neurotoxin treatments, prioritizing their needs, expectations, and perspectives on results. Post-procedural patient satisfaction remains a key assessment within this approach and has been widely studied. However, it is also important to consider how satisfaction levels impact on downstream actions. For example, when patients are unhappy with their treatment experience or outcomes, it is typically preferable that they express this directly to the injector—so that constructive solutions can be found—rather than engaging in more damaging behaviors, such as posting negative online reviews or discouraging others from seeking treatment.

To date, little research has been performed to assess the behaviors and actions that follow on from varying patient views on their own treatment. A recent US study examined negative online reviews of aesthetic procedures, and found that ineffectiveness and complications were the most frequently cited reasons for dissatisfaction.^1^ However, their analysis did not include individuals treated with cosmetic neurotoxins and did not examine positive experiences of treatment. A Korean study of patients who were dissatisfied with cosmetic, dental, or skin care/dietary services found that they were likely to engage in “active” behaviors as a result of their bad experience—such as switching service provider, negative word-of-mouth, or complaining to the treating healthcare professional (HCP).^2^ This research was not specific to aesthetic medicine and, again, made no comparison with behaviors following positive treatment experiences.

A number of validated instruments have been developed to assess patient-reported outcomes (PROs) with cosmetic neurotoxin treatment, and these tools have facilitated a deeper understanding of patient perspectives.^3-6^ However, PRO instruments do not capture specific behaviors in response to treatment.

Thus, there remains a need to better understand and differentiate patient actions in response to cosmetic neurotoxin treatments that they consider to be satisfactory versus those that are less satisfactory. This could help injectors to mitigate against more negative behaviors and might also facilitate enhanced patient–physician communication and improved “patient-centricity” during the post-treatment follow-up period.

The objective of the present study was to investigate the actions taken by patients following cosmetic neurotoxin injection, with regard to both their most positive and least positive treatment experiences.

## METHODS

### Study Design

This was an online, closed-ended, self-administered survey conducted among adults residing in 4 countries: Brazil, Canada, the United Kingdom, and the United States. Questionnaires were completed between September 26 and December 30, 2022.

The study was initiated only after IRB approval, provided by Advarra, Inc. (Columbia, MD). The work was conducted in accordance with consensus ethical principles derived from international guidelines (eg, the Council for International Organizations of Medical Sciences International Ethical Guidelines), International Council for Harmonisation/International Organization for Standardization good clinical practice (GCP) guidelines, and all other applicable laws and regulations. Respondents provided informed consent to take part in the study.

### Eligibility

Participants were required to meet the following criteria for inclusion: aged ≥18 years or over the local age of majority (whichever was higher); resident in 1 of the 4 participating countries; and had received 4 or more previous treatments with facial neurotoxin injections (using any available formulation) to temporarily improve the appearance of upper facial lines or wrinkles, at least one of which had been performed within the past 12 months.

### Survey Methodology

The survey was developed by the study sponsor (Allergan Aesthetics, an AbbVie Company, Irvine, CA) with input from The Harris Poll (Chicago, IL) and from a key opinion leader in health psychology (a principal investigator with expertise in PRO development, cosmetic neurotoxin products, and general best practices in survey design). It was then refined based on 3 cognitive debriefing interviews in the target population.

The final version of the survey was hosted by The Harris Poll on a single, secure, web-based platform to help ensure the uniformity of the survey instrument across countries, as well as the quality of data and comparability of the findings. It can be viewed as a text file in the Appendix.

Participants were sampled from online consumer panels comprised of adult members who had agreed to participate in survey-based research (not necessarily medically related). The study sponsor had no relationship with these panels. Potential participants were invited via e-mail or member portal to the secure website, where they were screened for eligibility; if they met the inclusion criteria, they then provided consent to continue and were able to complete the survey. Each invitation contained a unique password-protected survey link to ensure anonymity and prevent respondents from attempting it more than once. Participant confidentiality was maintained throughout.

Surveys were completed in each respondent's local language. All relevant instruments, including the screener, questionnaire, and consent form were developed in English and then translated into other languages (French in Canada and Portuguese in Brazil) by at least 2 independent professional translators who were native speakers of these languages.

Eligible respondents who completed the survey were required by design to answer all of the questions applicable to them. It was estimated to take around 15 min. As a token of appreciation, participants were offered the equivalent of USD 5 to 11 in online shopping vouchers for completion of the survey (the exact sum was country-specific).

The present paper describes the demographic and treatment characteristics of respondents, as well as their recollections of their most and least positive experiences of cosmetic neurotoxin treatment. It focuses particularly on levels of satisfaction with the process and outcomes, and the actions they engaged in after these experiences (eg, posting on social media, talking to friends and family, seeking further treatment, etc). In addition, exploratory statistical modeling was undertaken using responses from across the full breadth of the survey to assess the factors that were most predictive of undesirable behaviors as a result of participants' least positive experience.

### Statistical Analysis

Results were not weighted and are only representative of those individuals who participated in the research. The sample data were measured to be accurate to within ±2.4 percentage points using a Bayesian 95% confidence level.

Statistical analyses were performed using R and the R package “seminr”: Building and Estimating Structural Equation Models (version 2.3.2). Descriptive statistics are provided throughout, including mean and standard deviation for continuous variables, and percentages for categorical variables.

In addition, an exploratory, data-driven Partial Least Squares Structural Equation Modeling (PLS-SEM) analysis was performed. The aim was to examine the relationships and relative importance of various “latent variables” with regard to post-procedural behaviors following participants' least positive experience of treatment.

These latent variables were broad dimensions that took related results, observed directly from the survey (ie, similar metrics), and then summarized them into larger, indirectly inferred factors—such as patient experience, attitudes, social media habits, knowledge, demographics, and motivation. For example, one such hypothesized latent variable was the “Experience” dimension, which incorporated various measurements relating to participants' satisfaction with the treatment process and outcomes. Technically, these may be considered as proxies rather than true latent variables, functioning as approximations of the actual dimension.^7^ Nonetheless, we refer to them hereafter as latent variables. To ensure that they satisfactorily summarized their indicators, Cronbach's alpha score was used to measure their internal consistency (with an alpha score of <0.6 suggesting that the latent variable was inconsistent with some or all of its indicators, thus prompting revision). Latent variables were included in the PLS-SEM model and regressed to post-treatment outcomes. The model provided an indication of how strongly the variables predicted outcomes via an *R*-squared statistic, and coefficients of the model showed how important each one was in these predictions. Either a Johnson's epsilon statistic in case of regression or a sum of squared loadings was used to measure and scale the relative importance of the measured and latent variables in each step of the model.

## RESULTS

### Participant Characteristics

A total of 39,502 people were invited to participate, of whom 1612 eligible respondents completed the survey. Thus, the response rate was 4.1%. The majority were female (61%), and the mean age was 38.1 ± 9.6 years (Table 1). All had received at least 4 previous cosmetic neurotoxin treatments, and most (58%) had undergone 5 or more. Respondents were evenly distributed across the 4 participating countries.

**Table 1.** Participant Characteristics

Characteristic	Patients (*n* = 1612)
Sex, *n* (%)	
Female	980 (61)
Male	632 (39)
Age, years, mean ± SD	38.1 ± 9.6
Country, *n* (%)	
Brazil	400 (25)
Canada	403 (25)
United Kingdom	402 (25)
United States	407 (25)
Number of previous cosmetic neurotoxin treatments, *n* (%)	
Four	678 (42)
Five or more	934 (58)
Practitioner who typically performed their cosmetic neurotoxin treatments, *n* (%)	
Aesthetician	678 (42)
Doctor	580 (36)
Nurse, nurse practitioner, or PA	306 (19)
Other	16 (1)
Varied from injection to injection	32 (2)
Typical frequency of cosmetic neurotoxin treatments, *n* (%)	
Less than every 3 months	290 (18)
Every 3-4 months	516 (32)
Every 5-6 months	500 (31)
Every 7-9 months	161 (10)
Every 10-12 months	81 (5)
Once a year or less often	32 (2)
Only when I have an upcoming special event^a^	32 (2)
Other aesthetic procedures received, *n* (%)	
Dermal fillers	1064 (66)
Others^b^	1048 (65)

PA, physician assistant; SD, standard deviation.

^a^Such as a holiday, vacation, birthday, or wedding.

^b^Such as laser hair removal, microdermabrasion, or microneedling.

### Most and Least Positive Experiences

Participants were asked to reflect on their most and least positive experiences of cosmetic neurotoxin treatment. Their most positive experience was typically more recent, with 78% occurring in the past year compared with 51% of least positive experiences.

**Figure 1.**
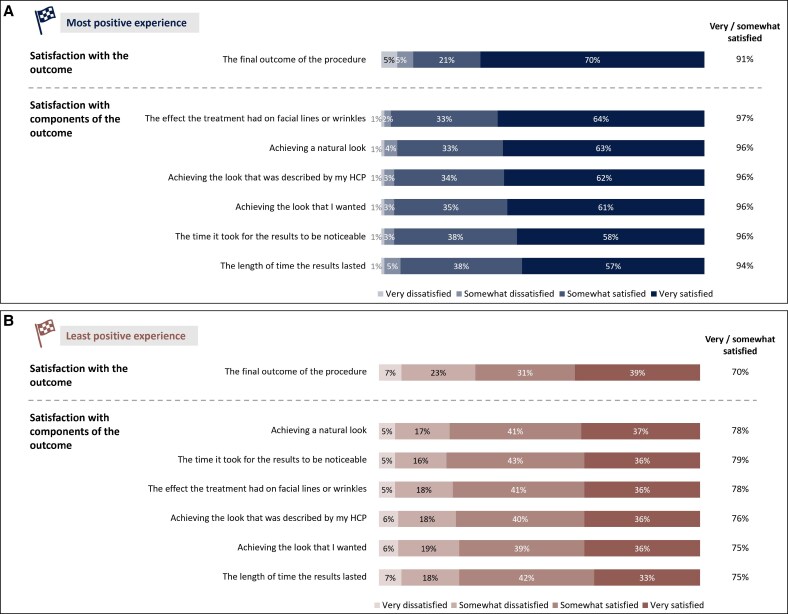
Satisfaction with outcomes in participants' most and least positive experiences with cosmetic neurotoxin treatment. Satisfaction with overall outcome and components of outcomes are shown for participants' (A) most positive, and (B) least positive experiences. *n* = 1612. HCP, healthcare professional.

With regard to their most positive experience, 91% of participants reported being “very” or “somewhat” satisfied with the overall outcome (Figure 1A). Satisfaction with specific components of the outcome, such as impact on facial lines, achieving a natural look, and duration of effect, ranged from 94% to 97% (Figure 1A). Additionally, 92% expressed satisfaction with the treatment *process* (eg, HCP skill level, friendliness, timeliness).

Regarding their least positive experience, this was not usually completely negative, with 70% of respondents reporting that they were very or somewhat satisfied with the outcome (Figure 1B). Satisfaction with components of the outcome ranged from 75% to 79%, while 75% said they were satisfied with the treatment process.

### Post-Treatment Actions

**Figure 2.**
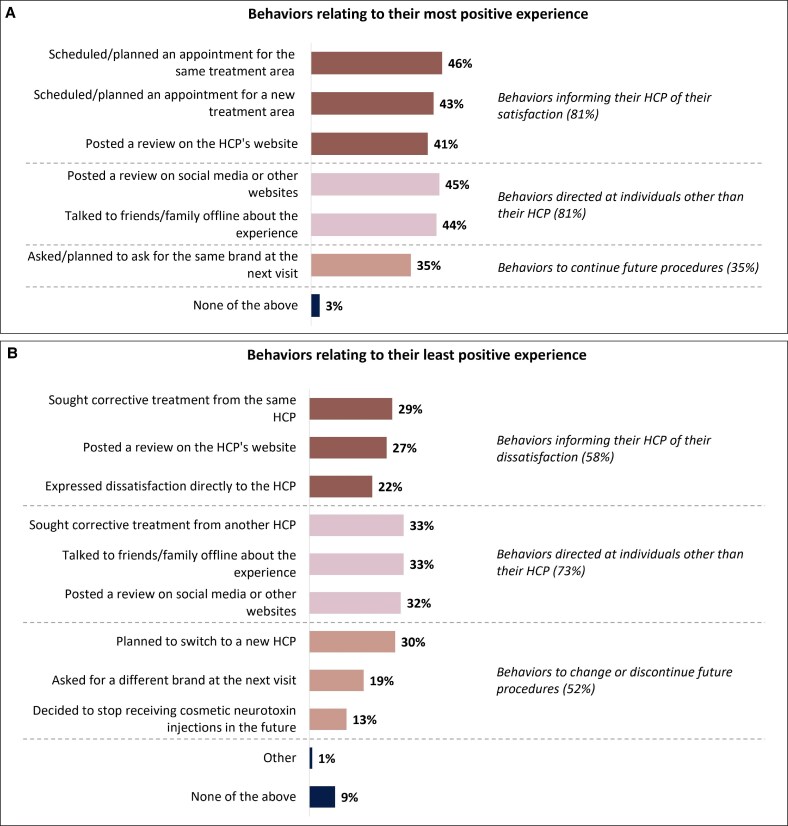
Participant actions after their most and least positive experiences with cosmetic neurotoxin treatment. Actions are shown for participants' (A) most positive, and (B) least positive experiences. Percentages may not add up to 100% because participants could choose multiple responses. *n* = 1612. HCP, healthcare professional.

After their most positive experience of cosmetic neurotoxin injection, 97% of participants said that they engaged in some sort of post-treatment action (Figure 2A). Most were directed toward their HCP (eg, scheduling another treatment or posting a review on the practitioner's website; 81%) or toward other people (eg, talking with friends and family or posting a review on social media; 81%).

After their least positive experience, 91% engaged in post-treatment actions (Figure 2B). However, just 58% directed these toward their HCP, and only 22% expressed dissatisfaction directly to the injector. More respondents (73%) took action to inform others, such as talking to friends and family (33%) or posting a negative review online (32%). In addition, 30% planned to switch HCP in future, and 13% decided to stop receiving cosmetic neurotoxin treatments altogether.

**Figure 3.**
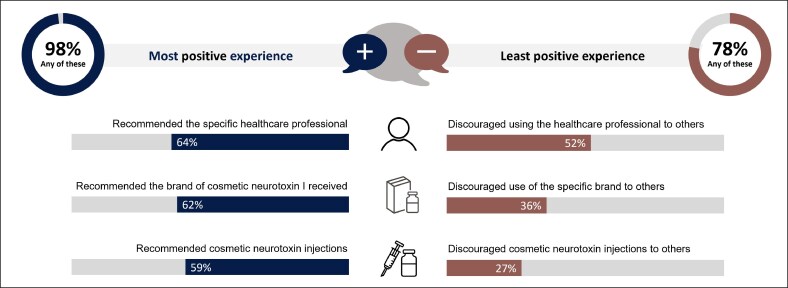
Recommendations and discouragements of those sharing details of their most or least positive treatment experience. Includes only patients who shared their experience in some way (ie, talked to friends and family or posted a review online). *n* = 1299 for the most positive experience; *n* = 1035 for the least positive experience. Percentages do not add up to 100% because participants could choose multiple responses.

Respondents who said they shared information with others (either by talking to friends and family or posting a review online) were then asked for further details (Figure 3). After their most positive experience, 64% said they had recommended the HCP and 59% had recommended cosmetic neurotoxin injections in general. After their least positive experience, 52% said they had discouraged others from using the same HCP, and 27% had discouraged cosmetic neurotoxin injections entirely.

Analysis at a national level showed no major differences in post-treatment actions across the 4 participating countries.

### Statistical Modeling of Least Positive Experiences

**Figure 4.**
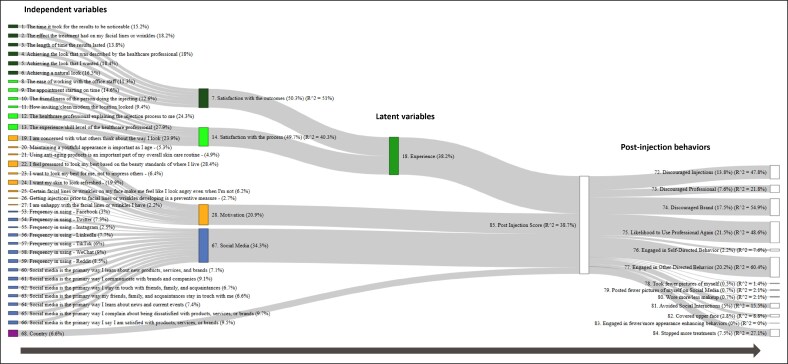
Sankey diagram summarizing the statistical modeling of participants' least positive experience of cosmetic neurotoxin treatment. Independent variables (those measured in the survey data) are represented on the far left. These are then brought together in the middle as (proxy) “latent variables”—composite factors that summarize various independent variables. Post-injection behaviors assessed in the survey are represented on the far right and are summarized by a latent variable called “post-injection score,” which was the ultimate target outcome. The exact relative importance is given in brackets in the labels and adds up to 100% in total. *R*-squared statistics (*R*^2^) are supplied where regression was used in the model. In addition, to aid interpretation, *R*-squared is also included on the post-injection behaviors to illustrate how much variance can be explained through the latent variable “post-injection score.” The node size (the height of the rectangles) is a measure of how relatively important each was in contributing to either a latent variable or in regressing to the next step in the model; these are not technically part of the structural equation model but rather transformations and ad hoc calculations that allow better visualization.

Exploratory modeling was used to examine factors affecting behavior following participants' least positive experience of cosmetic neurotoxin injection (Figure 4). Four statistically significant latent variables were implied. Expressed as a percentage, in descending order of impact on the model, these were the following: treatment experience (ie, satisfaction with the process and outcomes, 38.2%); social media usage (ie, frequency and likelihood of using these platforms for learning and communication, 34.3%); motivation (ie, concern around appearance and social pressures, 20.9%); and country of residence (6.6%).

## DISCUSSION

In this survey of 1612 individuals who had received multiple treatments with cosmetic neurotoxin, participants were more likely to inform their HCP of their satisfaction after the most positive experience compared with the least positive experience. Notably, only around a fifth said they proactively communicated directly with their HCP after their least positive experience. This aligns with previous research showing that negative healthcare experiences are rarely formally reported.^8^ However, more respondents said they informed *other people* about their least positive experience, either by talking to friends and family (around a third) or by posting online (also around a third). Often they specifically discouraged others from using their HCP or from seeking cosmetic neurotoxin injections altogether.

The recent Cosmetic Injectables Patient Experience Exploratory Study found that trust was the most important factor in choosing an HCP and that the reputation and recommendation of the practitioner was central to building such trust.^9-11^ This highlights the potential damage that negative reviews and poor word-of-mouth can do to an HCP's ability to build strong, trusting relationships with their patients.

**Figure 5.**
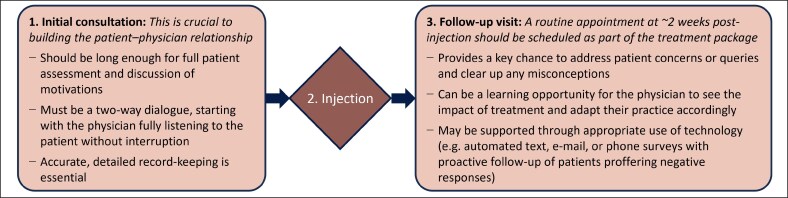
Key elements of a complete treatment protocol with cosmetic neurotoxin.

To mitigate this risk, effective communication is essential both before and after product administration. A complete treatment protocol should contain at least 3 key elements: an initial consultation, injection, and a follow-up visit (Figure 5).

Within this paradigm, the initial consultation should be long enough for a full patient assessment and comprehensive discussion of their motivations. Practitioners should start by listening without interruption and avoid cutting in too rapidly. Indeed, data suggest that clinicians eliciting patient concerns interrupt after a median of just 11 s,^12^ whereas patients typically need 2 min to fully express themselves.^13^ Individuals who feel listened to, reassured, and safe may be more likely to return if they have any subsequent concerns. Accurate, detailed record-keeping is also essential to this process.

With regard to follow up, a routine appointment should be scheduled around 2 weeks post-injection. Including it as part of the treatment package (ie, not charging separately) may help to encourage patients to return, and it can be done online if necessary. This appointment offers a chance to address lingering concerns or queries. Furthermore, given that perceived ineffectiveness and complications are common reasons for negative online reviews, proactive follow-up can help to clear up any misconceptions.^1^ In this regard, it is important to remember that neurotoxins are different from many other injectables in that results are not visible right away but rather develop over several days; some patients may therefore associate any adverse events over this period with the product, irrespective of whether there was a causal linkage. Furthermore, a well-structured follow-up appointment may be the only way for a practitioner to truly determine whether they have fulfilled the patient's expectations. As a result, injectors can take relevant learnings for improving their consultation and practical skills.

Technology may be deployed to support proactive follow-up, through the use of automated text, e-mail, or phone surveys to probe treatment experiences. Practitioners can then reach out personally to any patients submitting negative responses.

Failures of aftercare are often a key factor associated with poor patient ratings of aesthetic procedures.^14^  ^,15^ Proactive post-treatment follow-up can help to improve patient satisfaction and avert actions that are damaging to the practitioner. Moreover, this is not just a matter of managing reputational risk; it is also fundamental to good clinical practice. Indeed, a recent analysis of the ethical foundations of patient-centered care in aesthetic medicine stressed the importance of the patient–physician relationship and the value of follow-up.^16^

Validated PRO tools may be a valuable element of this process. They aid in the collection of patient experience data, and the results can be used to complement patient–physician discussions. Importantly, these instruments not only evaluate satisfaction with treatment but may also examine broader concepts like social confidence, self-esteem, feelings of attractiveness, and age appearance. Thus, they can facilitate a broad-ranging conversation on the impact of treatment.

Our exploratory statistical modeling identified factors that may predict “negative behaviors”—such as discouraging others to seek treatment—following less positive injection episodes. Unsurprisingly, respondents' own treatment experience was a key factor. This is an aspect that practitioners have significant control over and implies that optimizing the treatment process can mitigate the most damaging actions even among individuals who have negative overall experiences. Respondents' motivations and social media habits were also important. It makes sense that levels of engagement with social media would have an impact given that many of the post-injection behaviors assessed were online-related. Similarly, it seems logical that individuals who were particularly motivated by anxiety over what others think of their appearance would be more likely to engage in negative behaviors if results were suboptimal. These 2 factors are outside of HCP control. However, they do represent predictive features to look out for when there is concern over a patient's potential response to a negative treatment experience.

The present study had several important strengths. In particular, the respondent group was large and multinational, and there were no major differences in results between countries, suggesting the data may be broadly generalizable. Nonetheless, we must acknowledge that all participants were resident in North America, South America, or Europe, with no data collected in Asia or Africa. Post-treatment behaviors are likely to be somewhat culturally dependent, and hence caution should be exercised in extrapolating to other geographies. There are also other potential limitations of the work. For example, there is inherent potential for coverage error with a self-administered online survey, as well as possible recall bias due to the historical nature of the treatment experiences under consideration. In addition, the multiple-choice format might not have covered the full range of relevant actions undertaken by respondents. Although the response rate of 4.1% might appear low, the invitation to participate was sent to general online consumer panels and not specifically to aesthetic medicine patients; thus, relatively few would have been eligible (based on receiving 4 or more previous treatments with facial neurotoxin injections), and the response rate is therefore reasonable. It is notable that most participants' least positive experience with cosmetic neurotoxin was not entirely unsatisfactory; this is consistent with previous studies demonstrating high levels of overall satisfaction, but means that the results might not always have captured truly “negative” treatment episodes.^17^ Given that all respondents had received at least 4 rounds of injections with cosmetic neurotoxin, this would likely have been selective for individuals with more positive overall views of the treatment. Finally, the PLS-SEM methodology was exploratory and had no established underlying theoretical model, so the results of that analysis should be considered as suggestive rather than confirmatory.

## CONCLUSIONS

Participants in this international survey-based study were less likely to inform their HCP of their satisfaction level after their least positive experience of cosmetic neurotoxin treatment compared with the most positive. Thus, practitioners may often be unaware of dissatisfied individuals. The results underscore the importance of patient-centered care and consistent, proactive follow-up after treatment, in order to fully understand patient perspectives on outcomes—and facilitate communication around future options.

## Supplemental Material

This article contains supplemental material located online at www.asjopenforum.com.

## Supplementary Material

ojaf109_Supplementary_Data

## Data Availability

The data that support the findings of this study are available upon reasonable request. AbbVie is committed to responsible data sharing regarding the clinical trials we sponsor. This includes access to anonymized, individual, and trial-level data (analysis data sets), as well as other information (eg, protocols, clinical study reports, or analysis plans), as long as the trials are not part of an ongoing or planned regulatory submission. This includes requests for clinical trial data for unlicensed products and indications. These clinical trial data can be requested by any qualified researchers who engage in rigorous, independent, scientific research, and will be provided following review and approval of a research proposal, Statistical Analysis Plan (SAP), and execution of a Data Sharing Agreement (DSA). Data requests can be submitted at any time after approval in the US and Europe and after acceptance of this manuscript for publication. The data will be accessible for 12 months, with possible extensions considered. For more information on the process or to submit a request, visit the following link: https://vivli.org/ourmember/abbvie/ then select “Home”.

## References

[1] Watchmaker LE, Watchmaker JD, Callaghan D, Arndt KA, Dover JS. The unhappy cosmetic patient: lessons from unfavorable online reviews of minimally and noninvasive cosmetic procedures. Dermatol Surg. 2020;46:1191–1194. doi: 10.1097/DSS.0000000000002304 31876573

[2] Um KH, Lau AKW. Healthcare service failure: how dissatisfied patients respond to poor service quality. Int J Oper Prod Manag. 2018;38:1245–1270. doi: 10.1108/IJOPM-11-2016-0669

[3] Yaworsky A, Daniels S, Tully S, et al The impact of upper facial lines and psychological impact of crow's feet lines: content validation of the Facial Line Outcomes (FLO-11) Questionnaire. J Cosmet Dermatol. 2014;13:297–306. doi: 10.1111/jocd.12117 25399622

[4] Pompilus F, Burgess S, Hudgens S, Banderas B, Daniels S. Development and validation of a novel patient-reported treatment satisfaction measure for hyperfunctional facial lines: facial line satisfaction questionnaire. J Cosmet Dermatol. 2015;14:274–285. doi: 10.1111/jocd.12166 26264134

[5] Dayan S, Yoelin SG, De Boulle K, Garcia JK. The psychological impacts of upper facial lines: a qualitative, patient-centered study. Aesthet Surg J Open Forum. 2019;1:ojz015. doi: 10.1093/asjof/ojz015 33791609 PMC7671269

[6] FACE-Q Aesthetics . https://qportfolio.org/face-q/aesthetics. Accessed May 2025.

[7] Rigdon EE . Rethinking partial least squares path modeling: in praise of simple methods. Long Range Plann. 2012;45:341–358. doi: 10.1016/j.lrp.2012.09.010

[8] Wessel M, Lynøe N, Juth N, Helgesson G. The tip of an iceberg? A cross-sectional study of the general public's experiences of reporting healthcare complaints in Stockholm, Sweden. BMJ Open. 2012;2:e000489. doi: 10.1136/bmjopen-2011-000489 PMC3269049 22282539

[9] McDonald CB, Hart S, Liew S, Heydenrych I. The importance of patient mindset: cosmetic injectable patient experience exploratory study—part 1. Aesthet Surg J Open Forum. 2022;4:ojac043. doi: 10.1093/asjof/ojac043 35769690 PMC9225726

[10] McDonald CB, Heydenrych I. The importance of functional quality in patient satisfaction: cosmetic injectable patient experience exploratory study—part 2. Aesthet Surg J Open Forum. 2022;4:ojac044. doi: 10.1093/asjof/ojac044 35795885 PMC9252022

[11] McDonald CB, Heydenrych I. Factors influencing trust and trustworthiness: cosmetic injectable patient experience exploratory study (CIPEES)—part 3. Aesthet Surg J Open Forum. 2022;4:ojac082. doi: 10.1093/asjof/ojac082 36447650 PMC9687811

[12] Singh Ospina N, Phillips KA, Rodriguez-Gutierrez R, et al Eliciting the patient's agenda- secondary analysis of recorded clinical encounters. J Gen Intern Med. 2019;34:36–40. doi: 10.1007/s11606-018-4540-5 29968051 PMC6318197

[13] Langewitz W, Denz M, Keller A, Kiss A, Rüttimann S, Wössmer B. Spontaneous talking time at start of consultation in outpatient clinic: cohort study. BMJ. 2002;325:682–683. doi: 10.1136/bmj.325.7366.682 12351359 PMC126654

[14] Devgan LL, Klein EJ, Fox S, Ozturk T. Bifurcation of patient reviews: an analysis of trends in online ratings. Plast Reconstr Surg Glob Open. 2020;8:e2781. doi: 10.1097/GOX.0000000000002781 32440443 PMC7209897

[15] Bovenzi CD, Manges KA, Krein H, Heffelfinger R. Online ratings of facial plastic surgeons: worthwhile additions to conventional patient experience surveys. Facial Plast Surg Aesthet Med. 2021;23:78–89. doi: 10.1089/fpsam.2020.0049 32716653 PMC7994422

[16] Buttura da Prato E, Cartier H, Margara A, et al The ethical foundations of patient-centered care in aesthetic medicine. Philos Ethics Humanit Med. 2024;19:1. doi: 10.1186/s13010-024-00151-1 38317236 PMC10845625

[17] Cohen JL, Fagien S, Ogilvie P, et al High patient satisfaction for up to 6 months with onabotulinumtoxinA treatment for upper facial lines. Dermatol Surg. 2022;48:1191–1197. doi: 10.1097/DSS.0000000000003585 36342250 PMC9632938

