# Supplementary material for: The Patient Voice in Aesthetic Medicine: Findings From a Global Survey of Cosmetic Neurotoxin Patients
Source: Aesthet Surg J Open Forum. 2025 Oct 13;7:ojaf109. doi: 10.1093/asjof/ojaf109 (PMC12570879; doi:10.1093/asjof/ojaf109)
Supplement: ojaf109_Supplementary_Data [file ojaf109_supplementary_data.docx]

**Harris Insights & Analytics**

| **Client Name:** | [AESARA / AbbVie] | **Job Name:** | [Global Botox Toxin Survey] | |
| --- | --- | --- | --- | --- |
| **Wave No.**  **(srvyWave)** | 1 | **Job No. (dmJobNum)** | [P146039] | |
| **Landing Page Title (scrIntroTitle)** | [Thank you for agreeing to take this survey.] | | | |
| **LOI for ISQ section (isqLOI)** | | | | [15] (minutes) |
| **Digital Fingerprinting and Fraud Score [Imperium RelevantID] (dfOptions)** By default, surveys will terminate any respondents who fail both of these tests and is recommended for panel sample. For client sample or vendor sample, the termination of DF or Fraud Score can be turned off if desired.  *[SELECT CODES 1-3]* | | | | 1 Digital Fingerprinting/Fraud Score  2 Terminate DF Duplicate  3 Terminate DF Fraud Score |
| **Mode of survey (srvyMode)** | | | | 1 - Web |
| **Sample Sources (list/vlist)**  List sample sources used for the study; Client, HPOL, SSI, etc. | | | | Pure Profile, Quest Mindshare |
| **Respondent Type (srvyResp)**  Select ONE type of audience; if more than one select “Multiple” to indicate multiple audiences | | | | 1 – B2C |
| **Stagwell Company (srvyComp)** | | | | 1 – HIA (default) |
| **Other notes (i.e. Prior wave, programming notes, etc.)** | | | | None |

**SAMPLE PRELOAD AND SCREENING QUESTIONS**

**BASE: ALL RESPONDENTS**

**hCntry** HIDDEN: Country (from “co” URL variable)

14. Australia [English UK (uk)]

33. Brazil [Portuguese Brazil (portuguese_br)]

42. Canada [English UK (uk), French Canada (canadian)]

243. United Kingdom [English UK (uk)]

244. United States [English US (english)]

**BASE: ALL RESPONDENTS (srvyMode/1)**

**scrIntroTitle** [INSERT LANDING PAGE TITLE]

**scrIntro**

During the survey, please do not use your browser's *FORWARD* and *BACK* buttons. Instead, please always use the button below to move through the survey. Please be aware that once you've answered a question, you might not be able to go back and change your answer.

The progress bar below indicates approximately what portion of the survey you have completed.

Simply click on the button at the bottom of the page to begin the survey.

**BASE:   ALL RESPONDENTS (srvyMode/1)**

**dmConsent**

Thank you for agreeing to participate in this survey.  Your views are important to us and your answers will be kept in strict confidence. Please click here to read our privacy policy before agreeing to continue with the survey.

1.            I agree to continue

2.            I do not agree

[PN: IF dmConsent/2 TERMINATE IMMEDIATELY. INSERT "click here" AS HYPERLINK TO https://theharrispoll.com/privacy/]

**BASE: ALL RESPONDENTS**

**dmCntry** **[Country]** In which country or region do you currently reside?

[LIST CODES DISPLAYED IN ALPHABETICAL ORDER]

244. United States of America

14. Australia

33. Brazil

42. Canada

48. China

76. France

85. Germany

116. India

123. Italy

126. Japan

157. Mexico

215. Spain

243. United Kingdom

996. Other country

[PN: IF hCntry DOES NOT EQUAL dmCntry TERMINATE IMMEDIATELY.]

[PN: dmGen, dmAge PRESENTED ON SAME SCREEN.]

**BASE: ALL RESPONDENTS**

**dmGen [Gender]** Are you…?

1. Male

2. Female

**BASE: ALL RESPONDENTS**

**dmAge [Age]** What is your age?

[RANGE 0 -120]

|_|_|_|

[PN: IF NOT 18+ TERMINATE IMMEDIATELY.]

**BASE: ALL RESPONDENTS**

**finGen** HIDDEN: Gender (Final) **- USE FOR WEIGHTING ONLY, NOT FOR DELIVERABLES**

[IF dmGen/dmGenM=1, SELECT 1; IF dmGen/dmGenM=2, SELECT 2; IF dmGen/dmGenM<>1 OR 2 THEN RANDOMIZE 50/50]

1. Male

2. Female

**BASE: ALL RESPONDENTS**

**netAge** HIDDEN: Age (Net)

[COMPUTE AGE FROM dmAge]

1. 18-24

2. 25-34

3. 35-44

4. 45-54

5. 55-64

6. 65-74

7. 75+

**BASE: ALL RESPONDENTS**

**netGenAge** HIDDEN: Gender Age (Net)

[COMPUTE AGE/GENDER FROM finGen AND dmAge]

1. Male 18-24

2. Male 25-34

3. Male 35-44

4. Male 45-54

5. Male 55-64

6. Male 65+

7. Female 18-24

8. Female 25-34

9. Female 35-44

10. Female 45-54

11. Female 55-64

12. Female 65+

**BASE: ALL US RESPONDENTS (dmCntry/244)**

**dmStateUS [State (US)]** In what state or territory do you currently reside?

[DISPLAY IN DROP DOWN LIST]

1. Alabama

2. Alaska

3. Arizona

4. Arkansas

5. California

6. Colorado

7. Connecticut

8. Delaware

9. District of Columbia

10. Florida

11. Georgia

12. Hawaii

13. Idaho

14. Illinois

15. Indiana

16. Iowa

17. Kansas

18. Kentucky

19. Louisiana

20. Maine

21. Maryland

22. Massachusetts

23. Michigan

24. Minnesota

25. Mississippi

26. Missouri

27. Montana

28. Nebraska

29. Nevada

30. New Hampshire

31. New Jersey

32. New Mexico

33. New York

34. North Carolina

35. North Dakota

36. Ohio

37. Oklahoma

38. Oregon

39. Pennsylvania

40. Rhode Island

41. South Carolina

42. South Dakota

43. Tennessee

44. Texas

45. Utah

46. Vermont

47. Virginia

48. Washington

49. West Virginia

50. Wisconsin

51. Wyoming

52. American Samoa

53. Federated States of Micronesia

54. Guam

55. Marshall Islands

56. Northern Mariana Islands

57. Palau

58. Puerto Rico

59. Virgin Islands

**BASE: ALL CANADA RESPONDENTS (dmCntry/42)**

**dmRegionCA** **[Region (Canada)]** In which province or territory do you currently reside?

[DISPLAY IN DROP DOWN LIST - ALPHABETIZE CODES]

1. Alberta

2. British Columbia

3. Manitoba

4. New Brunswick

5. Newfoundland & Labrador

6. Northwest Territories

7. Nova Scotia

8. Nunavut

9. Ontario

10. Prince Edward Island

11. Quebec

12. Saskatchewan

13. Yukon

**BASE: ALL CANADA RESPONDENTS (dmCntry/42)**

**netRegionCA** HIDDEN: Region (Canada Net)

1. Atlantic (NL, PEI, NS, NB) [dmRegionCA/4,5,7,10]

2. Quebec [dmRegionCA/11]

3. Ontario [dmRegionCA/9]

4. Manitoba/Saskatchewan [dmRegionCA/3,12]

5. Alberta [dmRegionCA/1]

6. British Columbia [dmRegionCA/2]

7. Northwest (Nunavut, NWT, Y) [dmRegionCA/6,8,13]

**BASE: ALL RESPONDENTS**

**AOM** HIDDEN: Age of majority

[IF (dmAge/18+ and dmStateUS NE 1, 25, 28) OR (dmAge 19+ and dmStateUS/1,28) OR (dmAge 21+ and dmStateUS/25) OR (dmAge/18+ and dmCntry/14,33,243) OR (dmAge/18+ and dmRegionCA/1,3,4,9,10,11,12) OR (dmAge/19+ and dmRegionCA/2,5,6,7,8,13), SELECT 1; ELSE SELECT 2]

1. Yes
2. No

[PN: IF AOM NE/1, TERM IMMEDIATELY]

**BASE: ALL US RESPONDENTS (dmCntry/244)**

**netRegionUS** HIDDEN: Census Region (US Net)

1. Northeast [dmStateUS/7,20,22,30,40,46,31,33,39]

2. Midwest [dmStateUS/14,15,23,36,50,16,17,24,26,28,35,42]

3. South [dmStateUS/8,9,10,11,21,34,41,47,49,1,18,25,43,4,19,37,44]

4. West [dmStateUS/3,6,13,27,29,32,45,51,2,5,12,38,48]

5. Non-US State [dmStateUS/52-59]

[PN: IF netRegionUS/5, TERMINATE IMMEDIATELY]

**BASE: ALL US RESPONDENTS (dmCntry/244)**

**dmZipUS [Zip Code (US)]** What is your zip code?

[5 DIGITS – DISPLAY ERROR IF ZIP CODE IS NOT VALID]

[PN: INCLUDE ZIP/STATE MATCH SCREEN BUT DO NOT TERMINATE]

**BASE: ALL RESPONDENTS**

**Q101POPUP**: HIDDEN QUESTION: TEXT QUESTION FOR POPUPS

[PN: POP UP BOX USED FOR ALL INSTANCES INDICATED FOR “FACIAL NEUROTOXIN INJECTIONS”] :

Cosmetic neurotoxins are injectable medicines better known under brand names such as Botox Cosmetic®, Dysport®, Xeomin®, and Jeuveau®. Cosmetic neurotoxins work by causing facial muscles to relax, thereby smoothing wrinkles formed by frowning, squinting, and other facial expressions.

**BASE: ALL RESPONDENTS**

**Q101** Have you **ever** had any of the following cosmetic procedures?

[COLUMN; BANKED GRID]

1. Yes
2. No
3. Not sure

[ROWS; RANDOMIZE, ANCHOR CODE 3]

[PN: INCLUDE Q101POPUP FOR Q101/1]

1. Facial neurotoxin injections to temporarily improve the appearance of upper facial lines (e.g., Botox Cosmetic®, Dysport®, Xeomin®, Jeuveau®, etc.)
2. Dermal fillers (e.g., Juvederm®, Restylane®, Radiesse®, etc.)
3. Other procedures (e.g., laser hair removal, microdermabrasion, microneedling, etc.)

[PN: IF Q101/R1 = 2-3, TERMINATE IMMEDIATELY]

**BASE: HAVE HAD COSMETIC PROCEDURES (Q100c1 FOR ANY Q101r/1-3)**

**Q102** Thinking about the cosmetic procedures you’ve had, how many times have you gotten the following?

[COLUMNS; BANKED GRID]

1. 1 time
2. 2 times
3. 3 times
4. 4 times
5. 5+ times

[ROWS; ONLY DISPLAY THOSE SELECTED Q101c/1, SHOW IN SAME ORDER AS Q101]

[PN: INCLUDE Q101POPUP FOR Q102/1]

1. Facial neurotoxin injections to temporarily improve the appearance of upper facial lines (e.g., Botox Cosmetic®, Dysport®, Xeomin®, Jeuveau®, etc.)
2. Dermal fillers (e.g., Juvederm®, Restylane®, Radiesse®, etc.)
3. Other procedures (e.g., laser hair removal, microdermabrasion, microneedling, etc.)

[PN: IF Q102/R1 = 1-3, TERMINATE IMMEDIATELY]

**BASE: HAVE HAD COSMETIC PROCEDURES (Q100c1 FOR ANY Q100r/1-3)**

**Q103** You mentioned you have had the following type(s) of cosmetic procedures.

Please indicate when you had your **most recent** procedure for each.

[COLUMNS; BANKED GRID]

1. Within the past 3 months
2. 3 months to less than 6 months ago
3. 6 months to less than 1 year ago
4. 1 year to less than 2 years ago
5. 2 years to less than 5 years ago
6. 5 years or more ago

[ROWS;ONLY DISPLAY THOSE SELECTED Q101c/1, SHOW IN SAME ORDER AS Q101]

[PN: DISPLAY POP-UP BOX FOR FACIAL NEUROTOXIN INJECTIONS]

[PN: IF Q103/4-6, TERMINATE IMMEDIATELY]

**QUALIFICATION**

- Consent (dmConsent/1)
- Age of majority (18+ for US residents outside of Alabama, Nebraska, Mississippi; 19+ years of age in Alabama and Nebraska; 21+ years of age in Mississippi) (18+ for CAN residents in Alberta, Manitoba, New Brunswick, Ontario, Prince Edward Island, Quebec and Saskatchewan) (19+ for CAN residents in British Columbia, Newfoundland and Labrador, Northwest Territories, Nova Scotia, Nunavut, and Yukon) (18+ for AUS, UK, and BR residents) (AOM/1)
- Reside in the US, Australia, Brazil, Canada, or the UK (dmCntry/ 244, 243, 14, 33, 42)
- Had four or more cosmetic toxin injectable procedures ever (Q102/ 4-5)
- Most recent cosmetic toxin injectable procedure was within the past 12 months (Q103/ R1 = 1-3)

**QUOTAS**

- Total: n=2000
  - Australia: n=400
  - Brazil: n=400
  - Canada: n=400
  - UK: n=400
  - US: n=400

**QUOTAS BY VENDOR (TBD)**

**MAIN SURVEY**

**SECTION 1**

**BASE: ALL QUALIFIED RESPONDENTS**

**Q1** How much do you agree or disagree with each of the following?

[COLUMN; BANKED GRIDS]

1. Strongly disagree
2. Somewhat disagree
3. Somewhat agree
4. Strongly agree

[ROWS; RANDOMIZE]

[PN: INCLUDE Q101POPUP FOR Q1/8]

1. I am concerned with what others think about the way I look.
2. Maintaining a youthful appearance is important as I age.
3. Using anti-aging products is an important part of my overall skin care routine.
4. I feel pressured to look my best based on the beauty standards of where I live.
5. I want to look my best for me, not to impress others.
6. I want my skin to look refreshed.
7. Certain facial lines on my face make me feel like I look angry even when I’m not.
8. Getting cosmetic neurotoxin injections prior to facial lines or wrinkles developing is a preventive measure that helps maintain a youthful appearance.
9. I am unhappy with the facial lines I have.

**BASE: ALL QUALIFIED RESPONDENTS**

[PN: INCLUDE Q101POPUP FOR Q2]

**Q2** How old were you when you received your **first** cosmetic neurotoxin injections (e.g., Botox Cosmetic®, Dysport®, Xeomin®, Jeuveau®, etc.) to temporarily improve the appearance of upper facial lines?

[RANGE 18-dmAge]

|_|_| years old

**BASE: ALL QUALIFIED RESPONDENTS**

[PN: INCLUDE Q101POPUP FOR Q3]

**Q3** Which of the following best describes how often you typically receive cosmetic neurotoxin injections (e.g., Botox Cosmetic®, Dysport®, Xeomin®, Jeuveau®, etc.) to temporarily improve the appearance of upper facial lines?

[SINGLE RESPONSE]

1. Less than every 3 months
2. Every 3 to 4 months
3. Every 5 to 6 months
4. Every 7 to 9 months
5. Every 10 to 12 months
6. Once a year or less often
7. Only when I have an upcoming special event (e.g., holiday, vacation, birthday, wedding, etc.)

**BASE: ALL QUALIFIED RESPONDENTS**

[PN: INCLUDE Q101POPUP FOR Q4]

**Q4** Please indicate who **primarily** performs your cosmetic neurotoxin injections (e.g., Botox Cosmetic®, Dysport®, Xeomin®, Jeuveau®, etc.).

[SINGLE RESPONSE; RANDOMIZE; ANCHOR 96-98]

1. A doctor
2. A nurse, nurse practitioner, or physician assistant (PA)
3. An esthetician
4. Other
5. Not sure
6. It varies from injection to injection

**BASE: ALL QUALIFIED RESPONDENTS**

[PN: INCLUDE Q101POPUP FOR Q5]

**Q5** Before receiving your **first** cosmetic neurotoxin injections (e.g., Botox Cosmetic®, Dysport®, Xeomin®, Jeuveau®, etc.) to temporarily improve the appearance of upper facial lines, did you do any of the following?

[COLUMN; BANKED GRIDS]

1. Yes
2. No

[ROWS; RANDOMIZE; GROUP CODES 2-4 AND RANDOMIZE WITHIN]

[PN: INCLUDE Q101POPUP FOR Q5/R1-5]

1. Asked friends/family about their experience with cosmetic neurotoxin injections
2. Searched for/reviewed online information about **cosmetic neurotoxin injections** (e.g., watched videos, read reviews, read social media posts, etc.)
3. Searched for/reviewed online information about **different brands** of cosmetic neurotoxin injectables (e.g., watched videos, read reviews, read social media posts, etc.)
4. Searched for/reviewedonline information about **healthcare professionals** who perform cosmetic neurotoxin injections (e.g., watched videos, read reviews, read social media posts, etc.)
5. Spoke to healthcare professionals about cosmetic neurotoxin injections

**BASE: ALL QUALIFIED RESPONDENTS**

[PN: INCLUDE Q101POPUP FOR Q6]

**Q6** Before receiving your **first** cosmetic neurotoxin injections (e.g., Botox Cosmetic®, Dysport®, Xeomin®, Jeuveau®, etc.) to temporarily improve the appearance of upper facial lines, how knowledgeable did you feel about the following?

[COLUMN; BANKED GRIDS]

1. Not at all knowledgeable
2. Not very knowledgeable
3. Somewhat knowledgeable
4. Very knowledgeable

[ROWS; RANDOOMIZE; GROUP CODES 6-7 AND RANDOMIZE WITHIN]

[PN: INCLUDE Q101POPUP FOR Q6/R1-2]

1. Different brands of cosmetic neurotoxin
2. Potential risks or side effects of cosmetic neurotoxin injections
3. Level of discomfort to expect during the procedure
4. Length of time the procedure would take
5. What outcomes to expect (e.g., the expected look, how long it will take to see the results, how long it will last, etc.)
6. How long it would take to see final results
7. How long results would last
8. What the results would look like
9. What activities are allowed/not allowed for the first few days after the procedure

**SECTION 2: POSITIVE EXPERIENCE**

**[PN: ROTATE ORDER OF SECTION 2 “POSITIVE EXPERIENCE” AND SECTION 3 “NEGATIVE EXPERIENCE”]**

**BASE: ALL QUALIFIED RESPONDENTS**

[PN: INCLUDE Q101POPUP FOR INTRO1]

**INTRO1** For the next set of questions, we would like for you to think about when you had your **most positive** experience receiving a cosmetic neurotoxin injection (e.g., Botox Cosmetic®, Dysport®, Xeomin®, Jeuveau®, etc.) to temporarily improve the appearance of upper facial lines.

**BASE: ALL QUALIFIED RESPONDENTS**

[PN: INCLUDE Q101POPUP FOR Q7]

**Q7** How long ago was your **most positive** experience receiving a cosmetic neurotoxin injection (e.g., Botox Cosmetic®, Dysport®, Xeomin®, Jeuveau®, etc.) to temporarily improve the appearance of upper facial lines?

[SINGLE RESPONSE]

1. Less than 6 months ago
2. 6 to 11 months ago
3. 1 to 2 years ago
4. 3 to 4 years ago
5. 5 or more years ago

**BASE: ALL QUALIFIED RESPONDENTS**

[PN: INCLUDE Q101POPUP FOR Q8]

**Q8** Still thinking about your **most positive** experience, which of the following is true about the healthcare professional who performed that particular cosmetic neurotoxin injection (e.g., Botox Cosmetic®, Dysport®, Xeomin®, Jeuveau®, etc.)?

[SINGLE RESPONSE]

1. It was the first time I used that healthcare professional
2. I had used that healthcare professional once before
3. I had used that healthcare professional a few times before
4. I had used that healthcare professional many times before

**BASE: ALL QUALIFIED RESPONDENTS**

[PN: INCLUDE Q101POPUP FOR Q9]

**Q9** During your **most positive** experience, did you know the brand of cosmetic neurotoxin (e.g., Botox Cosmetic®, Dysport®, Xeomin®, Jeuveau®, etc.) that was injected to temporarily improve the appearance of upper facial lines ?

[SINGLE RESPONSE]

1. Yes
2. No

**BASE: KNOW THE BRAND OF COSMETIC NEUROTOXIN INJECTED (Q9/1)**

[PN: INCLUDE Q101POPUP FOR Q10]

**Q10** You indicated that you knew the brand of cometic neurotoxin (e.g., Botox Cosmetic®, Dysport®, Xeomin®, Jeuveau®, etc.) that was injected during your **most positive** experience. Which of the following describes your input on which brand to use?

[SINGLE RESPONSE; RANDOMIZE; ANCHOR CODE 97; HOLD CODES 2-3 TOGETHER AND ROTATE WITHIN]

[PN: INCLUDE Q101POPUP FOR Q10/R1]

1. I asked for a specific brand of cosmetic neurotoxin to be used
2. The healthcare professional told me which brand they would use
3. The healthcare professional and I discussed the brands available and made a joint decision
4. Not sure/can’t remember

**BASE: ALL QUALIFIED RESPONDENTS**

[PN: INCLUDE Q101POPUP FOR Q13]

**Q13** Thinking about your **most positive** experience receiving a cosmetic neurotoxin injection (e.g., Botox Cosmetic®, Dysport®, Xeomin®, Jeuveau®, etc.), please rate your satisfaction with the ***final outcome of the procedure.***

By “final outcome,” we mean the result you got after fully healing from the injection.

[SINGLE RESPONSE]

1. Very dissatisfied
2. Somewhat dissatisfied
3. Somewhat satisfied
4. Very satisfied

**BASE: ALL QUALIFIED RESPONDENTS**

[PN: INCLUDE Q101POPUP FOR Q14]

**Q14** Still thinking about your **most positive** experience receiving a cosmetic neurotoxin injection (e.g., Botox Cosmetic®, Dysport®, Xeomin®, Jeuveau®, etc.), please rate your satisfaction with each of the following aspects of the procedure outcomes.

[COLUMN; BANKED GRID]

1. Very dissatisfied
2. Somewhat dissatisfied
3. Somewhat satisfied
4. Very satisfied

[ROWS; RANDOMIZE; GROUP CODES 4-6 AND RANDOMIZE WITHIN]

1. The time it took for the results to be noticeable
2. The effect the treatment had on the facial lines
3. The length of time the results lasted
4. Achieving the look that was described by my healthcare professional
5. Achieving the look that I wanted
6. Achieving a natural look

**BASE: ALL QUALIFIED RESPONDENTS**

[PN: INCLUDE Q101POPUP FOR Q11]

**Q11** Thinking about your **most positive** experience receiving a cosmetic neurotoxin injection (e.g., Botox Cosmetic®, Dysport®, Xeomin®, Jeuveau®, etc.), please rate your satisfaction with the ***process.***

By “process,” we mean the clinic, the healthcare professional, the injection itself, etc.

[SINGLE RESPONSE]

1. Very dissatisfied
2. Somewhat dissatisfied
3. Somewhat satisfied
4. Very satisfied

**BASE: ALL QUALIFIED RESPONDENTS**

[PN: INCLUDE Q101POPUP FOR Q12]

**Q12** Still thinking about your **most positive** experience receiving a cosmetic neurotoxin injection (e.g., Botox Cosmetic®, Dysport®, Xeomin®, Jeuveau®, etc), please rate your satisfaction with each of the following aspects of the process.

[COLUMN; BANKED GRID]

1. Very dissatisfied
2. Somewhat dissatisfied
3. Somewhat satisfied
4. Very satisfied

[ROWS; RANDOMIZE]

1. The ease of working with the office staff
2. The appointment starting on time
3. The friendliness of the person doing the injecting
4. How inviting/clean/modern the location looked
5. The healthcare professional explaining the injection process to me (e.g., walking me through the treatment plan, what I should expect, etc.)
6. The experience/skill level of the healthcare professional

**BASE: ALL QUALIFIED RESPONDENTS**

**HIDDEN: HOLDS INSERTS FOR Q1**5

INSERT TEXT Q15/r6 BASED ON dmCntry

zQ15R6Text Q15 R6 Text

US (e.g., Facebook, Instagram, TikTok, Google, WeChat, RealSelf, Yelp, etc.)

AU (e.g., XXX, etc.)

BR (e.g., XXX, etc.)

UK (e.g., XXX, etc.)

CA (e.g., XXX, etc.)

**BASE: ALL QUALIFIED RESPONDENTS**

[PN: INCLUDE Q101POPUP FOR Q15]

**Q15** After your **most positive** experience receiving a cosmetic neurotoxin injection (e.g., Botox Cosmetic®, Dysport®, Xeomin®, Jeuveau®, etc), which of the following, if any, did you do? Please select all that apply.

[MULTI-RESPONSE; RANDOMIZE; GROUP CODES 1-2, 5-6 AND RANDOMIZE WITHIN; ANCHOR EXCLUSIVE CODE 97]

1. Scheduled/planned to make a next appointment for the **same** treatment area on my face
2. Scheduled/planned to make an appointment for a **new** treatment area on my face
3. Asked/planned to ask for the same brand at the next visit
4. Talked to friends/family offline about the experience
5. Posted a review on a **healthcare professional’s** website
6. Posted a review on **social media** **or other websites** [INSERT FROM zQ15R6]
7. Didn’t do any of the above

**BASE: SHARED EXPERIENCE IN SOME WAY (Q15/4-6)**

**Q16** When you talked to friends/family or posted a review, which of the following, if any, did you do? Please select all that apply.

[MULTI-RESPONSE; RANDOMIZE; ANCHOR EXCLUSIVE CODE 97; SHOW CODE 2 ONLY IF Q9/1]

[PN: INCLUDE Q101POPUP FOR Q16/R1-2]

1. Recommended cosmetic neurotoxin injections
2. Recommended the brand of cosmetic neurotoxin I received
3. Recommended the specific healthcare professional
4. None of these

**BASE: ALL QUALIFIED RESPONDENTS**

**Q17** Because of your **most positive** experience, which of the following, if any, did you do? Please select all that apply.

[MULTI-RESPONSE; RANDOMIZE; GROUP CODES 1-2, 6-7 AND RANDOMIZE WITHIN; ANCHOR EXCLUSIVE CODE 97]

1. Took more pictures of myself
2. Posted more pictures of myself on social media
3. Wore more/less makeup
4. Sought out more social interactions (e.g., going out with friends, etc.)
5. Exposed upper face (e.g., swept bangs/fringe aside, pulled hair back, etc.)
6. Engaged in **fewer** appearance-enhancing behaviors (e.g., dressing-up, styling hair, seeking out other cosmetic treatments, etc.)
7. Engaged in **more** appearance-enhancing behaviors (e.g., dressing up, styling hair, seeking out other cosmetic treatments, etc.)
8. Sought out other cosmetic treatments
9. None of these

**BASE: ALL QUALIFIED RESPONDENTS**

[PN: INCLUDE Q101POPUP FOR Q18]

**Q18** Thinking about your **most positive** experience receiving a cosmetic neurotoxin injection (e.g., Botox Cosmetic®, Dysport®, Xeomin®, Jeuveau®, etc), how likely are you to use the healthcare professional who performed that particular injection for other cosmetic neurotoxin injections in the future?

[SINGLE RESPONSE]

1. Not at all likely
2. Not very likely
3. Somewhat likely
4. Very likely

**SECTION 1: NEGATIVE EXPERIENCE**

**BASE: ALL QUALIFIED RESPONDENTS**

[PN: INCLUDE Q101POPUP FOR INTRO2]

**INTRO2** We would now like you to think about a time when you had your **least positive** experience receiving a cosmetic neurotoxin injection (e.g., Botox Cosmetic®, Dysport®, Xeomin®, Jeuveau®, etc) to temporarily improve the appearance of upper facial lines.

**BASE: ALL QUALIFIED RESPONDENTS**

[PN: INCLUDE Q101POPUP FOR Q19]

**Q19** How long ago was your **least positive** experience receiving a cosmetic neurotoxin injection (e.g., Botox Cosmetic®, Dysport®, Xeomin®, Jeuveau®, etc) to temporarily improve the appearance of upper facial lines?

[SINGLE RESPONSE]

1. Less than 6 months ago
2. 6 to 11 months ago
3. 1 to 2 years ago
4. 3 to 4 years ago
5. 5 or more years ago

**BASE: ALL QUALIFIED RESPONDENTS**

[PN: INCLUDE Q101POPUP FOR Q20]

**Q20** Still thinking about your **least positive** experience, which of the following is true about the healthcare professional who performed that particular cosmetic neurotoxin injection (e.g., Botox Cosmetic®, Dysport®, Xeomin®, Jeuveau®, etc)?

[SINGLE RESPONSE]

1. It was the first time I used that healthcare professional
2. I had used that healthcare professional once before
3. I had used that healthcare professional a few times before
4. I had used that healthcare professional many times before

**BASE: ALL QUALIFIED RESPONDENTS**

[PN: INCLUDE Q101POPUP FOR Q21]

**Q21** During your **least positive** experience, did you know the brand of cosmetic neurotoxin (e.g., Botox Cosmetic®, Dysport®, Xeomin®, Jeuveau®, etc.) that was injected to temporarily improve the appearance of upper facial lines ?

[SINGLE RESPONSE]

1. Yes
2. No

**BASE: KNOW THE BRAND OF COSMETIC NEUROTOXIN INJECTED (Q21/1)**

[PN: INCLUDE Q101POPUP FOR Q22]

**Q22** You indicated that you knew the brand of cosmetic neurotoxin (e.g., Botox Cosmetic®, Dysport®, Xeomin®, Jeuveau®, etc.) that was injected during your **least positive** experience. Which of the following describes your input on which brand to use?

[SINGLE RESPONSE; RANDOMIZE, HOLD CODES 2-3 TOGETHER AND ROTATE WITHIN; ANCHOR 97]

[PN: INCLUDE Q101POPUP FOR Q22/R1]

1. I asked for a specific brand of cosmetic neurotoxin to be used
2. The healthcare professional told me which brand they would use
3. The healthcare professional and I discussed the brands available and made a joint decision
4. Not sure/can’t remember

**BASE: ALL QUALIFIED RESPONDENTS**

[PN: INCLUDE Q101POPUP FOR Q25]

**Q25** Thinking about your **least positive** experience receiving a cosmetic neurotoxin injection (e.g., Botox Cosmetic®, Dysport®, Xeomin®, Jeuveau®, etc), please rate your satisfaction with the ***final outcome of the procedure.***

By “final outcome,” we mean the result you got after fully healing from the injection.

[SINGLE RESPONSE]

1. Very dissatisfied
2. Somewhat dissatisfied
3. Somewhat satisfied
4. Very satisfied

**BASE: ALL QUALIFIED RESPONDENTS**

[PN: INCLUDE Q101POPUP FOR Q26]

**Q26** Still thinking about your **least positive** experience receiving a cosmetic neurotoxin injection (e.g., Botox Cosmetic®, Dysport®, Xeomin®, Jeuveau®, etc), please rate your satisfaction with each of the following aspects of the procedure outcomes.

[COLUMN; BANKED GRIDS]

1. Very dissatisfied
2. Somewhat dissatisfied
3. Somewhat satisfied
4. Very satisfied

[ROWS; RANDOMIZE; GROUP CODES 4-6 AND RANDOMIZE WITHIN]

1. The time it took for the results to be noticeable
2. The effect the treatment had on my facial lines
3. The length of time the results lasted
4. Achieving the look that was described by the healthcare professional
5. Achieving the look that I wanted
6. Achieving a natural look

**BASE: ALL QUALIFIED RESPONDENTS**

[PN: INCLUDE Q101POPUP FOR Q23]

**Q23** Thinking about your **least positive** experience receiving a cosmetic neurotoxin injection (e.g., Botox Cosmetic®, Dysport®, Xeomin®, Jeuveau®, etc), please rate your satisfaction with the ***process.***

By “process,” we mean the clinic, the healthcare professional, the injection itself, etc.

[SINGLE RESPONSE]

1. Very dissatisfied
2. Somewhat dissatisfied
3. Somewhat satisfied
4. Very satisfied

**BASE: ALL QUALIFIED RESPONDENTS**

[PN: INCLUDE Q101POPUP FOR Q24]

**Q24** Still thinking about your **least positive** experience receiving a cosmetic neurotoxin injection (e.g., Botox Cosmetic®, Dysport®, Xeomin®, Jeuveau®, etc), please rate your satisfaction with each of the following aspects of the process.

[COLUMN; BANKED GRIDS]

1. Very dissatisfied
2. Somewhat dissatisfied
3. Somewhat satisfied
4. Very satisfied

[ROWS; RANDOMIZE]

1. The ease of working with the office staff
2. The appointment starting on time
3. The friendliness of the person doing the injecting
4. How inviting/clean/modern the location looked
5. The healthcare professional explaining the injection process to me (e.g., walking me through the treatment plan, what I should expect, etc.)
6. The experience/skill level of the healthcare professional

**BASE: ALL QUALIFIED RESPONDENTS**

**HIDDEN: HOLDS INSERTS FOR Q27**

INSERT TEXT Q27/r8 BASED ON dmCntry

zQ27R8Text Q27 R8 Text

US (e.g., Facebook, Instagram, TikTok, Google, WeChat, RealSelf, Yelp, etc.)

AU (e.g., XXX, etc.)

BR (e.g., XXX, etc.)

UK (e.g., XXX, etc.)

CA (e.g., XXX, etc.)

**BASE: ALL QUALIFIED RESPONDENTS**

[PN: INCLUDE Q101POPUP FOR Q27]

**Q27** After your **least positive** experience receiving a cosmetic neurotoxin injection (e.g., Botox Cosmetic®, Dysport®, Xeomin®, Jeuveau®, etc), which of the following, if any, did you do? Please select all that apply.

[MULTIPLE RESPONSE; RANDOMIZE; GROUP CODES 7-8, HOLD 2 and 4 AND RANDOMIZE WITHIN; ANCHOR 96,98; 98 EXLCUSIVE]

1. Complained to the healthcare professional
2. Sought corrective treatment from the **same healthcare professional**
3. Switched/planned to switch to a new healthcare professional
4. Sought corrective treatment from **another healthcare professional**
5. Asked for a different brand at the next visit
6. Talked to friends/family about the experience offline
7. Posted a review on the specific **healthcare professional’s** website
8. Posted a review on **social media or other websites** [INSERT TEXT FROM zQ27R8]
9. Decided to stop receiving cosmetic neurotoxin injections in the future
10. Other
11. Didn’t do anything

**BASE: SHARED EXPERIENCE IN SOME WAY (Q27/6-8)**

**Q28** When you talked to friends/family or posted a review, which of the following, if any, did you do? Please select all that apply.

[MULTIPLE RESPONSE; RANDOMIZE; ANCHOR EXCLUSIVE 98; SHOW CODE 3 ONLY IF Q21/1]

[PN: INCLUDE Q101POPUP FOR Q28/R1]

1. Discouraged cosmetic neurotoxin injections to others
2. Discouraged using the healthcare professional to others
3. Discouraged use of the specific brand to others
4. None of these

**BASE: ALL QUALIFIED RESPONDENTS**

**Q29** Because of your **least positive** experience, which of the following, if any, did you do? Please select all that apply.

[MULTIPLE RESPONSE; RANDOMIZE; GROUP CODES 6-7 AND RANDOMIZE WITHIN; ANCHOR EXCLUSIVE 98]

Took fewer pictures of myself

Posted fewer pictures of myself on social media

Wore more/less makeup

Avoided social interactions (e.g., called off work, postponed a special event, avoided social gatherings, etc.)

Tried to cover upper face (e.g., bangs/fringe on forehead, wearing hats or sunglasses, etc.)

1. Engaged in **fewer** appearance-enhancing behaviors (e.g., dressing-up, styling hair, seeking out other cosmetic treatments, etc.)
2. Engaged in **more** appearance-enhancing behaviors (e.g., dressing up, styling hair, seeking out other cosmetic treatments, etc.)
3. Decided to avoid other cosmetic treatments in the future
4. None of these

**BASE: ALL QUALIFIED RESPONDENTS**

**Q30** Thinking about your **least positive** experience receiving a cosmetic neurotoxin injection (e.g., Botox Cosmetic®, Dysport®, Xeomin®, Jeuveau®, etc), how likely are you to use this healthcare professional who performed that particular injection for other cosmetic neurotoxin injections in the future?

[SINGLE RESPONSE]

1. Not at all likely
2. Not very likely
3. Somewhat likely
4. Very likely

**SECTION 4**

**BASE: ALL QUALIFIED RESPONDENTS**

**INTRO3** Changing topics. We’d like to ask you just a few more questions that are not directly related to your experiences with cosmetic neurotoxin injections.

**BASE: ALL QUALIFIED RESPONDENTS**

**Q31** On a scale from 1 to 7 where ‘1’ means ‘strongly disagree’ and ‘7’ means ‘strongly agree’, please rate your agreement with each of the following statements.

[COLUMNS; BANKED GRID]

1. 1 – Strongly disagree
2. 2
3. 3
4. 4
5. 5
6. 6
7. 7 – Strongly agree

[ROWS; RANDOMIZE]

1. I often complain to the service provider when I’m dissatisfied with a service because I feel it is my duty to do so.
2. I am usually reluctant to complain to a service provider regardless of how bad a service is.
3. It sometimes feels good to get my dissatisfaction and frustration with the service out by complaining to the service provider.
4. By making complaints about unsatisfactory services, in the long run the quality of service will improve.
5. People have a responsibility to tell service providers when a service they purchase is unsatisfactory.
6. By complaining about defective services, I may prevent other consumers from experiencing the same problem.
7. I am less likely to return an unsatisfactory product than most people I know.

**BASE: ALL QUALIFIED RESPONDENTS**

**Q32** How frequently do you use each of the following social media platforms?

By “use”, we mean opening the app or website on your computer or other digital devices to view posts or create posts yourself.

[COLUMN; BANKED GRIDS]

1. Never
2. Less than once a month
3. Monthly
4. Weekly
5. Once a day
6. Several times a day

[ROWS; RANDOMIZE]

1. Facebook
2. Twitter
3. Instagram
4. LinkedIn
5. TikTok
6. WeChat
7. Reddit

**BASE: ALL QUALIFIED RESPONDENTS**

**Q33** How much do each of the following statements describe you **today**?

[COLUMN; BANKED GRIDS]

1. Does not describe me at all
2. Describes me a little
3. Describes me a fair amount
4. Describes me a lot
5. Describes me a great deal

[ROWS; RANDOMIZE; HOLD CODES 3-4 TOGETHER AND ROTATE WITHIN]

1. Social media is the primary way I learn about new products, services, and brands.
2. Social media is the primary way I communicate with brands and companies (e.g., direct message or tag brands and companies in posts).
3. Social media is the primary way I stay in touch with friends, family, and acquaintances.
4. Social media is the primary way my friends, family, and acquaintances stay in touch with me.
5. Social media is the primary way I learn about news and current events (e.g., following news accounts or reading news posts from friends and family).
6. Social media is the primary way I complain about being dissatisfied with products, services, and brands.
7. Social media is the primary way I say I am satisfied with products, services, and brands.

**DEMOGRAPHICS: US (dmCntry/244)**

**BASE: ALL US RESPONDENTS**

**dmEduUS [Education (US)]**  What is the highest level of education you have completed?

[SINGLE RESPONSEDISPLAY IN ONE COLUMN; GOING DOWN.]

1. Less than high school

2. Completed some high school

3. High school graduate

4. Job-specific training program(s) after high school

5. Some college, but no degree

6. Associate degree

7. Bachelor’s degree (such as B.A., B.S.)

8. Some graduate school, but no degree

9. Graduate degree (such as MBA, MS, M.D., Ph.D.)

**BASE: ALL US RESPONDENTS AND**

**netEduUS** Education (US Net)

1. Less than HS degree [dmEduUS/1,2]

2. HS degree to less than 4 year college degree [dmEduUS/3-6]

3. 4 year college degree or more [dmEduUS/7-9]

**BASE: ALL US RESPONDENTS 1**

**dmHhIncUS [Household Income (US)]**  How much total combined income did all members of your household earn before taxes last year?

*This includes money from jobs; net income from business, farm, or rent; pensions; dividends; interest; social security payments; and any other money income received by members of your household who are eighteen (18) years of age or older.*

[PROGRAMMER NOTE: DISPLAY IN ONE COLUMN; GOING DOWN.]

1. Less than $15,000

2. $15,000 to $24,999

3. $25,000 to $34,999

4. $35,000 to $49,999

5. $50,000 to $74,999

6. $75,000 to $99,999

7. $100,000 to $124,999

8. $125,000 to $149,999

9. $150,000 to $199,999

10. $200,000 to $249,999

11. $250,000 or more

[PROGRAMMER NOTE: INSERT “Why do we ask this question?” pop-up BELOW CHOICES]

**BASE: US AND**

**netHhIncUS** HIDDEN: Household Income (US Net)

1. Less than $15,000

2. $15,000-$24,999

3. $25,000-$34,999

4. $35,000-$49,999

5. $50,000-$74,999

6. $75,000-$99,999

7. $100,000 or more

**BASE: ALL US RESPONDENTS**

**dmHispUS [Hispanic Origin (US)]** Are you of Hispanic, Latino, or Spanish origin?

1. Yes

2. No

[PROGRAMMER NOTE: INSERT “Why do we ask this question?” pop-up BELOW CHOICES]

**BASE: ALL US RESPONDENTS**

**dmRaceMUS [Race-Multi (US)]** What is your race? Please select all that apply.

[PROGRAMMER NOTE: DISPLAY IN ONE COLUMN; MULTIPLE RESPONSE]

1. White

2. Black or African American

3. Native American or Alaskan Native

4. South Asian

5. Chinese

6. Korean

7. Japanese

8. Filipino

9. Arab/West Asian

12. Vietnamese

11. Other Asian

10. Native Hawaiian or Pacific Islander

13. Other race

[PROGRAMMER NOTE: INSERT “Why do we ask this question?” pop-up BELOW CHOICES]

**BASE:    ALL US RESPONDENTS**

**finRaceMUS**        HIDDEN: Race-Multi (US)   [FOR REPORTING; SINGLE RESPONSE]

14. Hispanic [dmHispUS/1]

1. White (only) [dmHispUS/NE 1 and dmRaceMUS/1 and dmRaceMUS/NE 2-13]

2. Black or African American (only) [dmHispUS/NE 1 and dmRaceMUS/2 and dmRaceMUS/NE 1,3-13]

3. Native American or Alaskan Native (only) [dmHispUS/NE 1 and dmRaceMUS/3 and dmRaceMUS/NE 1-2,4-13]

10. Native Hawaiian or Pacific Islander (only) [dmHispUS/NE 1 and dmRaceMUS/10 and dmRaceMUS/NE 1-9,11-13]

4. South Asian (only) [dmHispUS/NE 1 and dmRaceMUS/4 and dmRaceMUS/NE 1-3,5-13]

5. Chinese (only) [dmHispUS/NE 1 and dmRaceMUS/5 and dmRaceMUS/NE 1-4,6-13]

6. Korean (only) [dmHispUS/NE 1 and dmRaceMUS/6 and dmRaceMUS/NE 1-5,7-13]

7. Japanese (only) [dmHispUS/NE 1 and dmRaceMUS/7 and dmRaceMUS/NE 1-6,8-13]

8. Filipino (only) [dmHispUS/NE 1 and dmRaceMUS/8 and dmRaceMUS/NE 1-7,9-13]

9. Arab/West Asian (only) [dmHispUS/NE 1 and dmRaceMUS/9 and dmRaceMUS/NE 1-8,10-13]

15. Vietnamese (only) [dmHispUS/NE 1 and dmRaceMUS/12 and dmRaceMUS/NE 1-11,13]

11. Other Asian (only) [dmHispUS/NE 1 and dmRaceMUS/11 and dmRaceMUS/NE 1-10,12-13]

16. Asian (multiple) [dmHispUS/NE1 and (dmraceMUS/4-9,11,12/count >/=2) and dmRaceMUS/NE 1-3,10,13]

13. Some other race (only) [dmHispUS/NE 1 and dmRaceMUS/13 and dmRaceMUS/NE 1-12]

12. More than One Race [dmHispUS/NE 1 and All other dmRaceMUS/NE 99] (Excluding Asian multiple)

ASIAN (NET) [PN: THIS IS A NET IN THE SURVEY XML] = 4-9,15,11,16

**BASE: ALL US RESPONDENTS**

**netRaceMUS** HIDDEN: Race-Multi (US Net)

[PN: SINGLE RESPONSE]

1. Hispanic [dmHispUS/1]

2. Black only (not Hispanic) [dmHispUS/NE 1 and dmRaceMUS/2 and dmRaceMUS/NE 1,3-13]

3. Asian only (not Hispanic) [dmHispUS/NE 1 and dmRaceMUS/4-9,11-12 and dmRaceMUS/NE 1-3,10,13]

4. All Other (not Hispanic) [All other dmRaceMUS/NE 99]

**BASE:    ALL US RESPONDENTS**

**net2RaceMUS**    HIDDEN: Race-Multi (US Net 2 for RPR)

[PN: MULTIPLE RESPONSE]

1.             White only (not Hispanic) [dmHispUS/NE 1 and dmRaceMUS/1 and dmRaceMUS/NE 2-13]

2.             Hispanic  [dmHispUS/1]

3.             Black only (not Hispanic) [dmHispUS/NE 1 and dmRaceMUS/2 and dmRaceMUS/NE 1,3-13]

4.             Asian only (not Hispanic)  [dmHispUS/NE 1 and dmRaceMUS/4-9,11-12 and dmRaceMUS/NE 1-3,10,13]

5.             All Other (not Hispanic) [All other dmRaceMUS/NE 99]

6.             People of Color [all codes 2-5 above]

**DEMOGRAPHICS: CANADA (dmCntry/42)**

**BASE: CANADA RESPONDENT and language FOR WEIGHTING to be asked**

**dmFluencyCA**   **[Fluency (Canada)]** Which of the following languages can you speak well enough to conduct a conversation?

1.            English

2.            French

3.            Both English and French

4.            Neither English nor French

**BASE: ALL CANADA RESPONDENTS**

**dmEduCa** **[Education (Canada)]** Which of the following is the highest educational or professional qualification you have obtained?

1. Less than Secondary School (high school)
2. Completed some Secondary School (high school)
3. Graduated from Secondary School (high school) or equivalency certificate
4. Trade Certificate or Diploma
5. Certificate or Diploma from Community College, Institution, CEGEP
6. Teaching Certificate from Provincial Department of Education
7. Completed some University Study, but no Degree
8. University Certificate or Diploma below Bachelor Level
9. Bachelor or First Professional Degree
10. Graduate or Professional Degree above Bachelor Level

**BASE: ALL CANADA RESPONDENTS**

**netEduCA** HIDDEN: Education (Canada Net)

1. Below upper secondary [dmEduCA/1,2]

2. Upper secondary or post-secondary non-tertiary (vocational) [dmEduCA/3,4,7]

3. Tertiary (higher education) [dmEduCA/5,6,8-10]

**BASE:  ALL CANADA RESPONDENTS**

**dmRaceCA   [Race (Canada)]** Do you consider yourself…?

[SINGLE RESPONSE]

1. White

2. Black

3. First Nation/Native Canadian

4. South Asian

5. Chinese

6. Korean

7. Japanese

8. Other Asian

9. Filipino

10. Arab/West Asian

11. Pacific Islander

12. Mixed Race

13. Some other race

99. Prefer not to answer

**BASE: ALL CANADA RESPONDENTS**

**netRaceCA** HIDDEN: Race (Canada Net)

1. First Nation or Aboriginal [dmRaceCA/3]

2. Black [dmRaceCA/2]

3. South/Southeast Asian [dmRaceCA/4-8]

4. Other [dmRaceCA/9,10,11,12,13]

5. White [dmRaceCA/1]

**DEMOGRAPHICS: UK (dmCntry/243)**

**ALL UNITED KINGDOM RESPONDENTS**

**dmRegionUK [Region (UK)]** In which region or territory do you currently reside?

1. East of England

2. East Midlands

3. London

4. North East

5. North West

6. Northern Ireland

7. Scotland

8. South East

9. South West

10. Wales

11. West Midlands

12. Yorkshire and the Humber

**ALL UNITED KINGDOM RESPONDENTS**

**dmEduUK** **[Education (UK)]** Which of the following is the highest educational or professional qualification you have obtained?

1. Vocational qualifications (=NVQ1/NVQ2)

2. GCSE/O-Level/CSE

3. A-Level/'Scottish Higher or equivalent (=NVQ3)

4. Bachelor’s Degree or equivalent (=NVQ4)

5. Masters/PhD or equivalent

7. No formal qualifications

[NOTE: Code 6 “Other” should only be included in legacy QNRs]

**BASE: ALL UNITED KINGDOM RESPONDENTS**

**netEduUK** HIDDEN: UK EDUCATION

1. Below upper secondary [dmEduUK/7]

2. Upper secondary or post-secondary non-tertiary [dmEduUK/1,2,3]

3. Tertiary [dmEduUK/4,5]

*[Note: For dmEduUK/6 exclude or randomize, if legacy code]*

**AUSTRALIA**

**BASE: ALL AUstralia respondents**

**dmRegionAU [Region (Australia)]** In which state or territory do you currently reside?

1. Sydney

2. Other New South Wales

3. Melbourne

4. Other Victoria

5. Brisbane

6. Other Queensland

7. Adelaide

8. Other South Australia

9. Perth

10. Other Western Australia

11. Hobart

12. Other Tasmania

13. ACT

14. Northern Territory

**BASE: ALL AUSTRALIA RESPONDENTS**

**netRegionAU** HIDDEN: Region (Australia Net)

1. New South Wales [dmRegionAU/1,2]

2. Victoria [dmRegionAU/3,4]

3. Queensland [dmRegionAU/5,6]

4. South Australia [dmRegionAU/7,8]

5. Western Australia [dmRegionAU/9,10]

6. Tasmania [dmRegionAU/11,12]

7. Northern Territory [dmRegionAU/14]

8. Australian Capital Territory [dmRegionAU/13]

**BASE: ALL AUstralia respondents**

**dmEduAU [Education (Australia)]** What is the highest level of education you have completed or the highest degree you have received?

1. Year 11 or below

2. Year 12

3. Certificate I or II

4. Certificate III or IV

5. Certificate not further defined

6. Diploma/Advanced diploma

7. Bachelor degree or above

**BASE: ALL AUSTRALIA RESPONDENTS**

**netEduAU** HIDDEN: Education (Australia Net)

1. Below upper secondary [dmEduAU/1]

2. Upper secondary or post-secondary non-tertiary (vocational) [dmEduAU/2-5]

3. Tertiary (higher education) [dmEduAU/6,7]

**DEMOGRAPHICS: BRAZIL (dmCntry/33)**

**BASE: ALL BRAZIL RESPONDENTS**

**dmRegionBR** **[Region (Brazil)]** In which state do you currently reside?

[DISPLAY IN DROP DOWN LIST - ALPHABETIZE CODES]

1.          Acre

2.      Alagoas

3.          Amapa

4.          Amazonas

5.       Bahia

6.       Ceara

7.          Distrito Federal

8.       Espirito Santo

9.          Goias

10.       Maranhao

11.        Mato Grosso

12.        Mato Grosso do Sul

13.       Minas Gerais

14.        Para

15.       Paraiba

16.       Parana

17.       Pernambuco

18.       Piaui

19.       Rio de Janeiro

20.       Rio Grande do Norte

21.       Rio Grande do Sul

22.        Rondonia

23.       Roraima

24.      Santa Catarina

25.       Sao Paulo

26.       Sergipe

27.       Tocantins

**BASE: ALL BRAZIL RESPONDENTS**

**netRegionBR** HIDDEN: Region (Brazil Net)

1.     Central-West [dmRegionBR/7,9,11,12]

2.     North [dmRegionBR/1,3,4,14,22,23,27]

3.     Northeast [dmRegionBR/2,5,6,10,15,17,18,20,26]

4.     South [dmRegionBR/16,21,24]

5.     Southeast [dmRegionBR/8,13,19,25]

Education:

**BASE: ALL BRAZIL RESPONDENTS**

**dmEduBR [Education (Brazil)]** What is the highest level of education you have completed or the highest degree you have received?

1.          None

2.          Literate

3.          Incomplete Fundamental – fundamental I (1st series to 3rd series)

4.          Incomplete Fundamental - fundamental II (4th series to 7th series)

5.          Complete Fundamental

6.          Complete Secondary education (Ensino Medio)

7.          University (Superior)

8.          Post graduate (Masters, Doctorate or Post Doctorate)

**BASE: ALL BRAZIL RESPONDENTS**

**net1EduBR** HIDDEN: Education (Brazil Net for *GenPop* weighting)

1. Less than upper secondary [dmEduBR/1-5]
2. Upper secondary or more [dmEduBR/6]
3. Tertiary (Higher Education) [dmEduBR/7,8]

**BASE: ALL BRAZIL RESPONDENTS**

**net2EduBR** HIDDEN: Education (Brazil Net for *OnlinePop* weighting)

1. Less than secondary [dmEduBR/1-5]

2. Secondary or more [dmEduBR/6-8]

Socio-economic status *(GenPop only, not OnlinePop)*

**BASE: ALL BRAZIL RESPONDENTS**

**dmEduHoHBR [Education – Head of Household (Brazil)]** What is the highest level of education the head of your household has completed or the highest degree he/she has received?

1.          None

2.          Literate

3.          Incomplete Fundamental – fundamental I (1st series to 3rd series)

4.          Incomplete Fundamental - fundamental II (4th series to 7th series)

5.          Complete Fundamental

6.          Complete Secondary education (Ensino Medio)

7.          University (Superior)

8.          Post graduate (Masters, Doctorate or Post Doctorate)

**DEMOGRAPHICS (ALL QUALIFIED COMPLETES)**

**BASE: ALL RESPONDENTS AND**

**dmMarStat [Marital Status]** What is your current marital status?

1. Never married

2. Married or civil union

3. Divorced

4. Separated

5. Widowed

6. Living with partner

**BASE: ALL RESPONDENTS AND**

**netMarStat** HIDDEN: Marital Status (Net)

1. Never married [dmMarStat/1]

2. Married/Living with partner [dmMarStat/2,6]

3. Divorced/Separated/Widowed [dmMarStat/3,4,5]

**BASE: ALL RESPONDENTS**

**dmEmploy [Employment Status]** Which of the following best describes your employment status?

[SINGLE RESPONSE]

1. Employed full time

2. Employed part time

3. Self-employed full time

4. Self-employed part time

5. Not employed, but looking for work

6. Not employed and not looking for work

7. Not employed, unable to work due to a disability or illness

8. Retired

9. Student

10. Stay-at-home spouse or partner

**BASE: ALL RESPONDENTS**

**netEmploy** HIDDEN: Employment Status (Net)

1. Employed (FT, PT or Self) [dmEmploy/1,2,3,4]

2. All Other (Unemployed, Retired, Student, Homemaker, etc.) [dmEmploy/5-10]

**BASE: ALL RESPONDENTS**

**dmUrban [Size of Place]** Which of the following best describes the area in which you currently reside?

1. Urban/Metro

2. Rural/Non-Metro

**ISQ – IN SURVEY QUALITY METRICS**

| **ISQ Metrics to be used (isqOptions)**  *There are 6 primary types of quality checks listed here. Select which of the 13 total ISQ checks will be used. Codes 1 and 2 are on by default.* | **[PN: SELECT CODES 1, 2, 98, 99]**  **Codes 1-14 are evaluated together, and the respondent is terminated at the end of the survey if 2 or more are failed.**  1 Under Minimum Length of Interview (isqLOI)  2 Incorrect Response to Respondent Instruction Default (isqRI1)  3 Incorrect Response to Respondent Instruction Custom (isqRI2)  9 Incorrect Response to Respondent Instruction Custom (isqRI3)  10 Incorrect Response to Respondent Instruction Custom (isqRI4)  11 Incorrect Response to Respondent Instruction Custom (isqRI5)  4 Straight-Line Grid Question(s) (isqSL)  6 Less Than 5 Words and/or No Vowels at OE (isqOE)  7 Illogical Response to Survey Questions #1 (isqIC1)  8 Illogical Response to Survey Questions #2 (isqIC2)  12 Illogical Response to Survey Questions #3 (isqIC3)  13 Illogical Response to Survey Questions #4 (isqIC4)  14 Illogical Response to Survey Questions #5 (isqIC5)  **Codes 97-99 are terminated immediately when failed.**  97 [FUTURE USE]  98 Honeypot (white text question - isqHPT)  99 Real Answer Verbatim Evaluation (specify question from list below or enter existing Q#): [1] |
| --- | --- |
| **1. LOI (qtime) and LOI check (isqLOI)** | **LOI Check** = Response equivalents for the longest survey path converted to estimated LOI.  The minimum acceptable LOI is 30% of estimated LOI. |
| **2. Respondent Instruction Test Result Default (isqRI1)** | A standard respondent instruction question is shown by default before the demos or wherever indicated. |
| **3. Respondent Instruction Test Result Custom (isqRI2)**  *Define custom logic for a 2^nd^ respondent instruction test. For example, a question or an attribute in a grid that must be answered a certain way.* | **[PN: ENTER CUSTOM LOGIC HERE]** |
| **9. Respondent Instruction Test Result Custom (isqRI3)**  *Define custom logic for a 3^rd^ respondent instruction test. For example, a question or an attribute in a grid that must be answered a certain way.* | **[PN: ENTER CUSTOM LOGIC HERE]** |
| **10. Respondent Instruction Test Result Custom (isqRI4)**  *Define custom logic for a 4^th^ respondent instruction test. For example, a question or an attribute in a grid that must be answered a certain way.* | **[PN: ENTER CUSTOM LOGIC HERE]** |
| **11. Respondent Instruction Test Result Custom (isqRI5)**  *Define custom logic for a 5^th^ respondent instruction test. For example, a question or an attribute in a grid that must be answered a certain way.* | **[PN: ENTER CUSTOM LOGIC HERE]** |
| **4. Straight-lining Grid Check (isqSL)** *No longer limited to selecting 1 grid, define your custom logic here. You can identify 1 or multiple grids where straight-lining is suspicious. For example, if respondent straight lines 3 out of 5 grids listed, this check fails. Identifying grids with 15 attributes or more is recommended, but grids with minimum of 5 attributes will work.* | [**PN: ENTER CUSTOM LOGIC HERE FOR A STRAIGHTLINING CHECK]** |
| **6. Incomplete Response at Open End (isqOE)** *Identify an open end question. If the respondent provides less than 5 words and/or no vowels, including ‘y’, it will be flagged.)* | **[PN:** **CHECK FOR LESS THAN 5 WORDS/NO VOWELS OE RESPONSE AT QXXX]** |
| **7. Illogical Choice Combination #1 (isqIC1)** *Define custom logic for an illogical choice combination. This could include a low incidence activity in a screening question, a contradictory response based on a prior question, or a respondent selects or a respondent says he has been in his current job for 20 years, but that he is 35.* | [**PN: ENTER CUSTOM LOGIC HERE FOR AN ILLOGICAL RESPONSE CHECK]** |
| **8. Illogical Choice Combination #2 (isqIC2)** *Define custom logic for an illogical choice combination. This could include a low incidence activity in a screening question, a contradictory response based on a prior question, or a respondent selects or a respondent says he has been in his current job for 20 years, but that he is 35.* | [**PN: ENTER CUSTOM LOGIC HERE FOR AN ILLOGICAL RESPONSE CHECK]** |
| **12. Illogical Choice Combination #3 (isqIC3)** *Define custom logic for an illogical choice combination. This could include a low incidence activity in a screening question, a contradictory response based on a prior question, or a respondent selects or a respondent says he has been in his current job for 20 years, but that he is 35.* | [**PN: ENTER CUSTOM LOGIC HERE FOR AN ILLOGICAL RESPONSE CHECK]** |
| **13. Illogical Choice Combination #4 (isqIC4)** *Define custom logic for an illogical choice combination. This could include a low incidence activity in a screening question, a contradictory response based on a prior question, or a respondent selects or a respondent says he has been in his current job for 20 years, but that he is 35.* | [**PN: ENTER CUSTOM LOGIC HERE FOR AN ILLOGICAL RESPONSE CHECK]** |
| **14. Illogical Choice Combination #5 (isqIC5)** *Define custom logic for an illogical choice combination. This could include a low incidence activity in a screening question, a contradictory response based on a prior question, or a respondent selects or a respondent says he has been in his current job for 20 years, but that he is 35.* | [**PN: ENTER CUSTOM LOGIC HERE FOR AN ILLOGICAL RESPONSE CHECK]** |
| **Quality Checks that Failed (isqFail)** | 1 Under Minimum Length of Interview (isqLOI)  2 Incorrect Response to Respondent Instruction Default (isqRI1)  3 Incorrect Response to Respondent Instruction Custom (isqRI2)  9 Incorrect Response to Respondent Instruction Custom (isqRI3)  10 Incorrect Response to Respondent Instruction Custom (isqRI4)  11 Incorrect Response to Respondent Instruction Custom (isqRI5)  4 Straight-Line Grid Question(s) (isqSL)  6 Less Than 5 Words and/or No Vowels at OE (isqOE)  7 Illogical Response to Survey Questions #1 (isqIC1)  8 Illogical Response to Survey Questions #2 (isqIC2)  12 Illogical Response to Survey Questions #3 (isqIC3)  13 Illogical Response to Survey Questions #4 (isqIC4)  14 Illogical Response to Survey Questions #5 (isqIC5) |
| **Number of Quality Checks Failed (isqFailNum)**  *FM/RESEARCHER: MINIMUM ISQ FAILURES IS SET TO 2* | 0 Failed 0  1 Failed 1  2 Failed 2  3 Failed 3  4 Failed 4  5 Failed 5  6 Failed 6  7 Failed 7  8 Failed 8  9 Failed 9  10 Failed 10  11 Failed 11  12 Failed 12  13 Failed 13 |
| **Notes** | The order of evaluation is:  Immediate Terms = Dupe, DF, Honeypot, RA  All the remaining ISQ checks are evaluated at the end of the survey. |

**REAL ANSWER – RESPONDENT VERBATIM RESPONSE QUALITY CHECK**

**BASE: QUALIFIED RESPONDENT AND REAL ANSWER-SUPPORTED LANGUAGE**

**raTmplQText** HIDDEN, TEMPLATE QUESTION TEXT FOR REAL ANSWER TEST

[PN: ENTER QUESTION CODE IN GRID ABOVE, IF USING EXISTING SURVEY QUESTION ENTER Q# IN GRID ABOVE.]

1. What is your best childhood memory? Please be as descriptive as possible.

2. What do you feel most proud of? Please be as descriptive as possible.

3. If you could travel anywhere, where would you go and why? Please be as descriptive as possible.

4. What is your favorite hobby and why? Please be as descriptive as possible.

5. What is your favorite meal and why? Please be as descriptive as possible.

6. Who is your favorite historical figure and why? Please be as descriptive as possible.

7. Where do you see yourself in 5 years? Please be as descriptive as possible.

8. What is a specific goal that you would like to meet over the next 3 years? Please be as descriptive as possible.

**BASE: QUALIFIED RESPONDENT, TEMPLATE QUESTION TEXT USED AND REAL ANSWER-SUPPORTED LANGUAGE**

**raTmpl** REAL ANSWER RESPONDENT QUESTION

Changing topics...

[PN: INSERT TEXT FROM raTmplQText] [TEXT BOX]

**BASE: ALL RESPONDENTS**

**vTerm** VIRTUAL: Terminate Status

[TERMINATE CODES ARE CUSTOM BASED ON EACH STUDY]

**BASE: ALL RESPONDENTS**

**vTermAlt** VIRTUAL: Terminate Status Alternate

1. Survey Terminate (Immediate)

2. Survey Terminate (After Demos)

3. DF Duplicate

4. DF Fraud Score Failure

5. Real Answer Failure

6. ISQ Failure [Includes Honeypot]

7. Recode: ISQ Failure

8. Overquota (Immediate)

9. Overquota (After Demos)

10. Recode: Survey Error

11. Recode: Other Reason

**BASE: ALL RESPONDENTS**

**status** VIRTUAL: Respondent Status (system variable)

1. Terminated

2. Overquota

3. Qualified

4. Partial (Suspend)

[“Why do we ask this question?” POP-UP TEXT BELOW]

Collecting this information from all survey respondents is important to more accurately represent people from different backgrounds and experiences in the final results of the survey.

We understand that you might be concerned about sharing this information. Results are reported as averages for groups of people, and not for any specific person. The responses you provide are kept completely confidential.

Thank you for your participation.
